# Maternal dietary methionine restriction alters hepatic expression of one-carbon metabolism and epigenetic mechanism genes in the ducklings

**DOI:** 10.1186/s12864-022-09066-7

**Published:** 2022-12-12

**Authors:** Aurélie Sécula, Lisa E. Bluy, Hervé Chapuis, Agnès Bonnet, Anne Collin, Laure Gress, Alexis Cornuez, Xavier Martin, Loys Bodin, Cécile M. D. Bonnefont, Mireille Morisson

**Affiliations:** 1 GenPhySE, Université de Toulouse, INRAE, ENVT, F-31326 Castanet Tolosan, France; 2 INRAE, Université de Tours, BOA, 37380 Nouzilly, France; 3 UEPFG INRA Bordeaux-Aquitaine (Unité Expérimentale Palmipèdes à Foie Gras), Domaine d’Artiguères 1076, route de Haut Mauco, F-40280 Benquet, France

**Keywords:** Duck, Methyl donor, Nutritional programming, Differentially expressed genes, Avian

## Abstract

**Background:**

Embryonic and fetal development is very susceptible to the availability of nutrients that can interfere with the setting of epigenomes, thus modifying the main metabolic pathways and impacting the health and phenotypes of the future individual. We have previously reported that a 38% reduction of the methyl donor methionine in the diet of 30 female ducks reduced the body weight of their 180 mule ducklings compared to that of 190 ducklings from 30 control females. The maternal methionine-restricted diet also altered plasmatic parameters in 30 of their ducklings when compared to that of 30 ducklings from the control group. Thus, their plasma glucose and triglyceride concentrations were higher while their free fatty acid level and alanine transaminase activity were decreased. Moreover, the hepatic transcript level of 16 genes involved in pathways related to energy metabolism was significantly different between the two groups of ducklings. In the present work, we continued studying the liver of these newly hatched ducklings to explore the impact of the maternal dietary methionine restriction on the hepatic transcript level of 70 genes mostly involved in one-carbon metabolism and epigenetic mechanisms.

**Results:**

Among the 12 genes (*SHMT1*, *GART*, *ATIC*, *FTCD*, *MSRA*, *CBS*, *CTH*, *AHCYL1*, *HSBP1*, *DNMT3*, *HDAC9* and *EZH2*) identified as differentially expressed between the two maternal diet groups (*p*-value < 0.05), 3 of them were involved in epigenetic mechanisms. Ten other studied genes (*MTR*, *GLRX*, *MTHFR*, *AHCY*, *ADK*, *PRDM2*, *EEF1A1*, *ESR1*, *PLAGL1*, and *WNT11*) tended to be differently expressed (0.05 < *p*-value < 0.10). Moreover, the maternal dietary methionine restriction altered the number and nature of correlations between expression levels of differential genes for one-carbon metabolism and epigenetic mechanisms, expression levels of differential genes for energy metabolism, and phenotypic traits of ducklings.

**Conclusion:**

This avian model showed that the maternal dietary methionine restriction impacted both the mRNA abundance of 22 genes involved in one-carbon metabolism or epigenetic mechanisms and the mRNA abundance of 16 genes involved in energy metabolism in the liver of the newly hatched offspring, in line with the previously observed changes in their phenotypic traits.

**Supplementary Information:**

The online version contains supplementary material available at 10.1186/s12864-022-09066-7.

## Background

During early life, epigenetic mechanisms -the most frequently cited of which are DNA methylation, histone post-translational modifications and non-coding RNAs- contribute to the establishment of different epigenomes that allow the differentiation of cell lineages and embryonic tissues. The nutritional status encountered during embryonic life interferes with the establishment of these epigenomes, resulting in gene expression changes, influencing key metabolic pathways and affecting the health and phenotypes of the offspring even in the adult stage. This phenomenon is known as nutritional programming [1–4]. Thus, the effects of maternal nutrition on offspring metabolic diseases or production performances have been largely documented and reviewed in mammals [5–11] as well as in poultry [12–14].

Among the nutrients that can affect epigenetic mechanisms, methyl donors are widely cited as they impact the availability of S-adenosylmethionine (SAM). An inadequate amount of SAM leads to a lower availability of methyl groups altering DNA methylation. It may for instance modify methylation of differentially methylated regions (DMR) or cytosines within CpG rich regions located upstream genes, in the promotor regions. SAM is also involved in the post-translational methylation of histones as well as in methylation of miRNA, consequently modulating their maturation and activity [15, 16]. SAM is thus a key link between the one-carbon metabolism pathway and epigenetic mechanisms in the nutritional programming process [17–23]. Consequently, methyl donors such as folate, choline, betaine or methionine, are considered as dietary factors that can reshape the cellular epigenomes during embryonic life and may thus alter the phenotypes in the adult life [21, 24, 25]. Moreover, a number of research studies in birds have reported that methyl donor availability plays critical roles in hepatic carbohydrate and lipid metabolism. For example, *in ovo* injection of betaine has been reported to affect hepatic cholesterol metabolism in newly hatched chicks through epigenetic mechanisms, including DNA and histone methylations [26]. It also showed a protective effect on corticosterone-induced hepatic steatosis, which was associated with increased expression of *PPARα* and *CPT1α* [27]. Finally, when betaine was administrated to hens instead of being injected into eggs, it changed hepatic expression of a number of genes in chicks from betaine-fed hens [28, 29]. Indeed, betaine is a methyl donor that feeds the methionine cycle, which in turn provides methyl groups to methyltransferases, some of which methylating DNA or histones. Thus, this chain of events can ultimately lead to the deregulation of carbohydrate and lipid metabolism genes in the liver [24].

Encouraged by this literature, and particularly by the work on chicken mentioned above, we wanted to know whether a methyl donor deficiency in the female duck diet could have an impact on the liver metabolism of the offspring. Our question was posed in the context of fatty liver production so called “foie gras” production, in which the fatty livers come from the male mule ducks that are the offspring of female common ducks (*Anas platyrhynchos*) and Muscovy drakes (*Cairina moschata*) [30]. We therefore developed a model to determine whether a methionine deficiency applied to the female ducks could alter the liver characteristics of the offspring. In a first step, we use the model to explore the molecular mechanisms and metabolic pathways impacted by maternal diet in duckling livers at hatching. In a next step, we will study the phenotypic traits and hepatic expression levels of the same genes on the adult offspring (14 weeks of age) -after a 12 days period of overfeeding- for which a reduction in the “foie gras” production was observed (unpublished data). Thus, we have already reported the effects of a reduced level of dietary methionine (Met) on laying performances of female common ducks and its impacts on the phenotypes of their newly hatched mule ducklings [31]. Briefly, the restricted group of dams received Met-restricted diets (R group) containing 0.25% of Met whereas the control group received control diets (C group) containing 0.40% of Met during the growing and laying periods, from 10 to 51 weeks of age. Newly hatched ducklings from the R group had lower body weights. In addition, several of their plasma parameters were affected such as glucose and triglyceride (TG) concentrations which were higher while free fatty acid (FFA) level as well as alanine transaminase (ALT) activity were decreased. These observations suggested an alteration in hepatic energy metabolism in newly hatched ducklings from Met-restricted dams. This hypothesis was thus investigated by analyzing the hepatic mRNA abundance of 100 genes involved in energy metabolism and identifying 16 of them as differentially expressed in the liver of ducklings from Met-restricted dams compared to ducklings from control dams [32]. Most of them are involved in different pathways related to energy metabolism such as glycolysis, lipogenesis or electron transport whereas others are nuclear receptors such as *PPARGC1B*, *PPARG* and *RXRA*.

The objective of the present study was to investigate if the maternal dietary methionine deficiency could have also affected the transcription level of genes involved in one-carbon metabolism and epigenetic mechanisms in the duckling liver. For that, we compared the mRNA abundance of 70 genes mostly involved in one-carbon metabolism and epigenetic mechanisms in the liver of the ducklings from the two groups. We also enlightened correlations between the transcription level of genes involved in one-carbon metabolism, epigenetic mechanisms, and energy metabolism.

## Results

The hypothesis studied was that the maternal Met deficiency affected the hepatic transcription level of genes involved in one-carbon metabolism and epigenetic mechanisms in ducklings from the Met-restricted dams. We thus sought to compare the normalized relative expression of 70 genes (Additional Table 2) related to these metabolisms in 38 livers from ducklings of the two groups (50% of males and 50% of females). However, 8 of the 70 genes studied and 2 of the 38 liver cDNA samples showed more that 25% of missing expression data and were removed from the study. Moreover, another cDNA sample was considered as outlier and was also removed from the data set (see Methods section). The 35 remaining liver cDNA samples are from 10 male and 8 female ducklings from the Met-restricted dams and 9 male and 8 female ducklings from the control dams. The ducklings that are the offspring of the dams fed the Met-restricted and the control diets are subsequently designed as R and C groups, respectively.

### Maternal met deficiency downregulated genes involved in one-carbon metabolism and epigenetic mechanisms

For the 62 studied genes, the normalized and transformed relative expressions were used to look for differences in gene expression between samples from the two diet groups i.e. R group versus C group (Additional Table 1; Diet *P*-value (BH)). The effect of the sex of the ducklings (Sex *P*-value (BH)) as well as the interaction between sex and diet effects were also evaluated (Sex*Diet *P*-value (BH)). For each gene, least square means and standard deviations are given for the two groups of maternal diet (R and C groups), for the two sexes of the ducklings and for the four subgroups of ducklings i.e. males and females from the R group (MR and FR) and males and females from the C group (MC and FC).

**Fig. 1:**
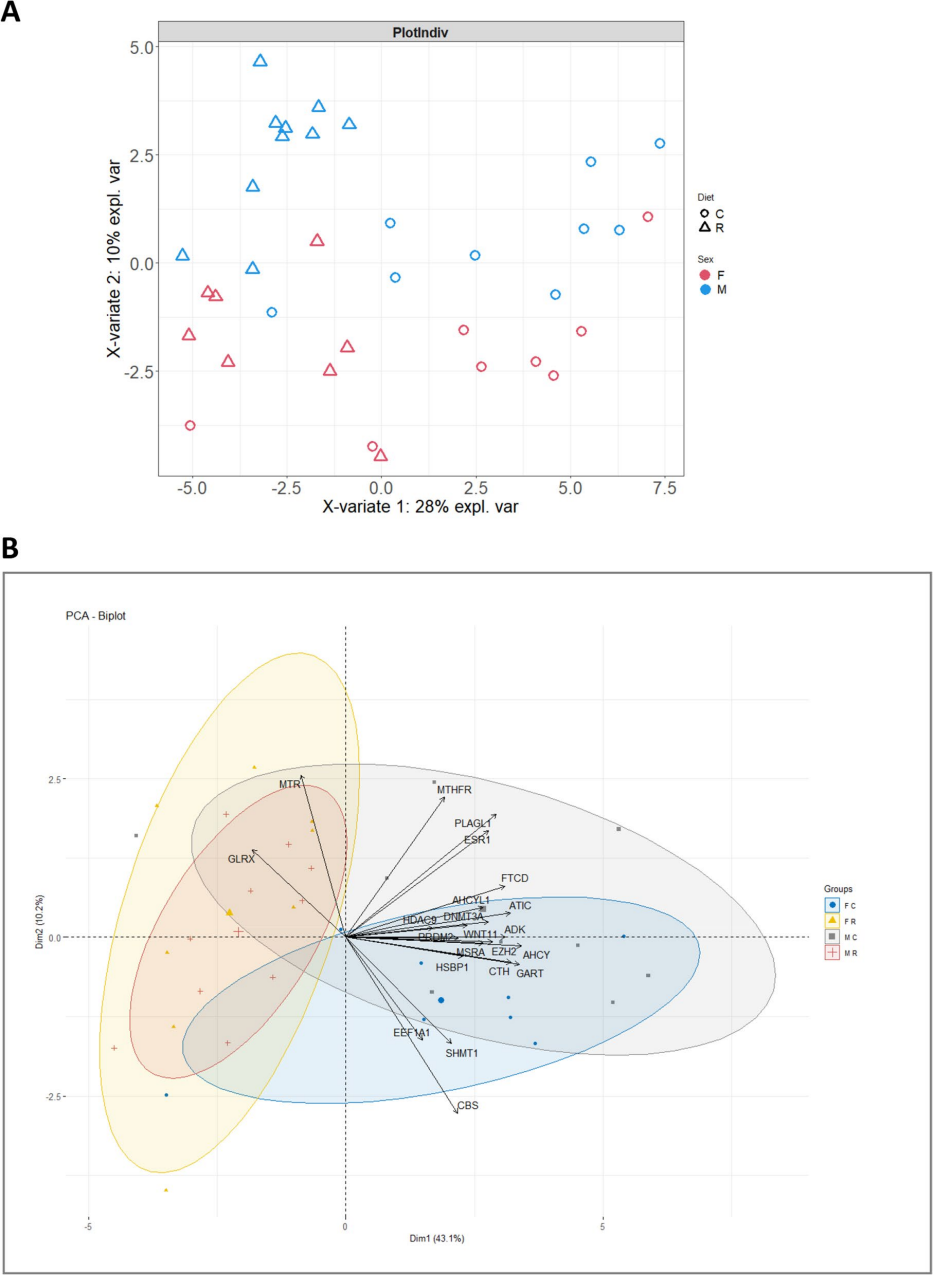
Exploratory data analyses. A Score plot of a PLS performed on the data of the 62 studied genes: The ducklings from R group and C group are represented with triangles and circles, respectively. The females are in red and the males in blue. The two latent variables summarized respectively 28% (horizontal axis) and 10% (vertical axis) of the whole variability. B Biplot of a PCA performed on the data of the 22 differential genes: The male ducklings from the R group (MR) and the C group (MC) are represented in red crosses and grey squares, respectively, and the females from the R group (FR) and the C group (FC) are in yellow triangles and blue circles, respectively. The first principal component (horizontal axis) explained 43.1% of the whole variability and discriminated the samples according to the diet received by the dams (R groups on the left side versus C groups on the right side). The second principal component (vertical axis) explained 10.2% of the whole variability and slightly discriminated the two sexes in the C group only. In addition, the correlation circles showed correlations between the 22 differential genes and the two main principal components and show an opposite regulation pattern of *GLRX* and *MTR* when compared to the 20 other differential genes. For both Fig. 1A and B, the qqnorm transformed normalized relative expressions were used

The Table 1 is extracted from the Additional Table 1 and describes the genes showing a significant difference imputable either to the maternal diet or to the sex of the duckling. No gene showed a significant interaction between the maternal diet effect and the sex effect (Sex*Diet *p*-value (BH) > 0.1). One-third of the 62 studied genes were found either to be differently expressed genes (12 DEGs with a Diet *p*-value (BH) <  0.05) or to tend to be differently expressed between the two diet groups (10 genes with a 0.05 < Diet p-value (BH) <  0.10). These 22 genes were further called “differential genes”. They were all down-regulated in the R group samples when compared to C group samples, except *GLRX* and *MTR* that were upregulated. In addition, 5 genes showed a significant effect of the sex of the ducklings (*GLRX*, *BHMT*, *BHMT2*, *DHFR* and *MAT2* with a Sex *p*-value (BH) <  0.05 noted in bold in Table 1) and 2 genes (*RBBP4* and *HNF4A*, in bold with a delta) tended to be differentially expressed (0.05 < Sex p-value (BH) <  0.1). The score plot (distribution of individuals) of the PLS (Partial Least Squares) method performed on the 62 studied genes along the 2 first latent variables showed that the samples were first separated by the maternal diet on the horizontal axis and then by the sex of the ducklings on the vertical axis (Fig. 1A). The two latent variables summarized respectively 28% (horizontal axis) and 10% (vertical axis) of the whole variability. Thus, the samples were separated not only according to the maternal diet but also according to the sex of the ducklings thus defining four subgroups i.e. males from the R group (MR), females from the R group (FR), males from the C group (MC) and females from the C group (FC), in accordance to the genes with a sex effect (Table 1).

**Table 1:** Differentially expressed genes in the liver of ducklings

Gene	R group LsMeans ± SD (*n* = 18)	C group LsMeans ± SD (*n* = 17)	Males LsMeans ± SD (*n* = 19)	Females LsMeans ± SD (*n* = 16)	Diet *P*-value (BH)	Sex *P*-value (BH)	Sex*Diet *P*-value (BH)
*ATIC*	− 0.57 ± 0.21	0.51 ± 0.22	0.22 ± 0.20	− 0.27 ± 0.23	< 0.01	0.54	0.95
*HDAC9*	−0.71 ± 0.26	0.66 ± 0.27	0.13 ± 0.25	−0.18 ± 0.26	< 0.01	0.77	0.52
*EZH2*	−0.49 ± 0.20	0.53 ± 0.21	0.00 ± 0.19	0.05 ± 0.21	< 0.01	0.94	0.58
*SHMT1*	− 0.52 ± 0.21	0.56 ± 0.22	0.10 ± 0.21	−0.06 ± 0.22	< 0.01	0.94	0.95
*HSBP1*	−0.52 ± 0.20	0.50 ± 0.21	0.23 ± 0.20	−0.25 ± 0.21	0.01	0.51	0.95
*GART*	−0.56 ± 0.25	0.53 ± 0.29	0.10 ± 0.25	−0.13 ± 0.26	0.01	0.93	0.98
*AHCYL1*	−0.47 ± 0.22	0.48 ± 0.22	0.10 ± 0.21	−0.08 ± 0.23	0.01	0.94	0.97
*CBS*	−0.53 ± 0.22	0.51 ± 0.24	−0.05 ± 0.22	0.03 ± 0.23	0.01	0.94	0.52
*MSRA*	−0.44 ± 0.21	0.50 ± 0.21	−0.02 ± 0.20	0.08 ± 0.23	0.01	0.94	0.52
*DNMT3A*	−0.43 ± 0.22	0.55 ± 0.25	0.26 ± 0.24	−0.14 ± 0.23	0.02	0.77	0.95
*CTH*	−0.48 ± 0.24	0.46 ± 0.22	0.10 ± 0.22	−0.13 ± 0.24	0.02	0.94	0.87
*FTCD*	−0.55 ± 0.30	0.52 ± 0.35	0.03 ± 0.29	−0.06 ± 0.30	0.02	0.94	0.95
***GLRX***	0.27 ± 0.18	−0.37 ± 0.18	0.52 ± 0.17	−0.63 ± 0.19	0.07	**< 0.01**	0.52
*ADK*	−0.42 ± 0.25	0.37 ± 0.28	0.27 ± 0.25	−0.32 ± 0.26	0.07	0.31	0.95
*MTR*	0.33 ± 0.22	−0.40 ± 0.22	0.25 ± 0.21	−0.31 ± 0.23	0.07	0.31	0.95
*AHCY*	−0.40 ± 0.25	0.41 ± 0.29	0.20 ± 0.26	−0.19 ± 0.26	0.07	0.77	0.95
*PLAGL1*	−0.37 ± 0.23	0.37 ± 0.23	0.14 ± 0.22	−0.14 ± 0.24	0.07	0.93	0.95
*ESR1*	−0.32 ± 0.22	0.37 ± 0.23	−0.05 ± 0.21	0.09 ± 0.23	0.07	0.94	0.59
*MTHFR*	−0.58 ± 0.34	0.43 ± 0.38	−0.01 ± 0.32	−0.15 ± 0.34	0.07	0.94	0.95
*PRDM2*	−0.37 ± 0.24	0.37 ± 0.24	0.00 ± 0.22	0.00 ± 0.25	0.07	0.95	0.85
*WNT11*	−0.32 ± 0.23	0.39 ± 0.26	−0.01 ± 0.23	0.08 ± 0.26	0.08	0.94	0.81
*EEF1A1*	−0.40 ± 0.30	0.46 ± 0.34	0.00 ± 0.29	0.06 ± 0.31	0.09	0.94	0.95
***BHMT***	0.06 ± 0.23	− 0.18 ± 0.25	0.56 ± 0.22	−0.67 ± 0.24	0.70	**< 0.01**	0.52
***BHMT2***	0.04 ± 0.26	−0.10 ± 0.29	0.51 ± 0.25	−0.57 ± 0.26	0.90	**< 0.01**	0.55
***DHFR***	−0.08 ± 0.17	−0.08 ± 0.18	0.70 ± 0.16	− 0.86 ± 0.17	0.97	**< 0.01**	0.78
***MAT2***	−0.31 ± 0.23	0.19 ± 0.25	0.37 ± 0.22	−0.49 ± 0.24	0.30	**0.04**	0.52
***RBBP4*** ^δ^	0.17 ± 0.20	− 0.27 ± 0.20	0.33 ± 0.19	−0.43 ± 0.21	0.18	**0.06** ^δ^	0.52
***HNF4A*** ^δ^	−0.12 ± 0.22	0.00 ± 0.23	0.36 ± 0.22	−0.47 ± 0.24	0.90	**0.08** ^δ^	0.58

Numbers, LS-Means, and standard deviations of the gene expression as well as the significance of the effects are given. For the diet effect, the 12 genes differently expressed between the two diet groups (Diet *p*-value (BH) <  0.05) are listed in the first part of the table whereas the 10 ones which tend to be differently expressed (Diet p-value (BH) <  0.10) are listed in the second part of the table. In addition, for the duckling sex effect, the 5 genes showing a significant effect (Sex *P*-value (BH) <  0.05) are in bold and the 2 genes which tended to be differentially expressed (Sex P-value (BH) <  0.1) are in bold noted with a delta (^δ^). No gene showed a significant interaction between the maternal diet effect and the duckling sex effect (Sex*Diet P-value (BH)). The data used were the qqnorm transformed normalized relative expressions

The Fig. 1B shows the biplot of a PCA (Principal Component Analysis) performed only on the 22 differential genes i.e. the 12 DEGs (Diet *p*-value (BH) <  0.05) and the 10 ones which tended to be differentially expressed for the diet effect (0.05 < Diet p-value (BH) <  0.1). The first principal component (horizontal axis) explained 43.1% of the whole variability and discriminated the samples according to the diet received by the dams (R groups versus C groups). The second principal component (vertical axis) explained 10.2% of the whole variability and slightly discriminated the two sexes in the C group only. In addition, the correlation circle showed correlations between the 22 differential genes and the two main principal components and confirmed the opposite regulation pattern of *GLRX* and *MTR* when compared to the other differential genes, as reported in Table 1.

### The altered correlations between the differential genes and the phenotypic traits reinforced evidences for an altered liver metabolism in ducklings from dams fed met-deficient diet

**Fig. 2:**
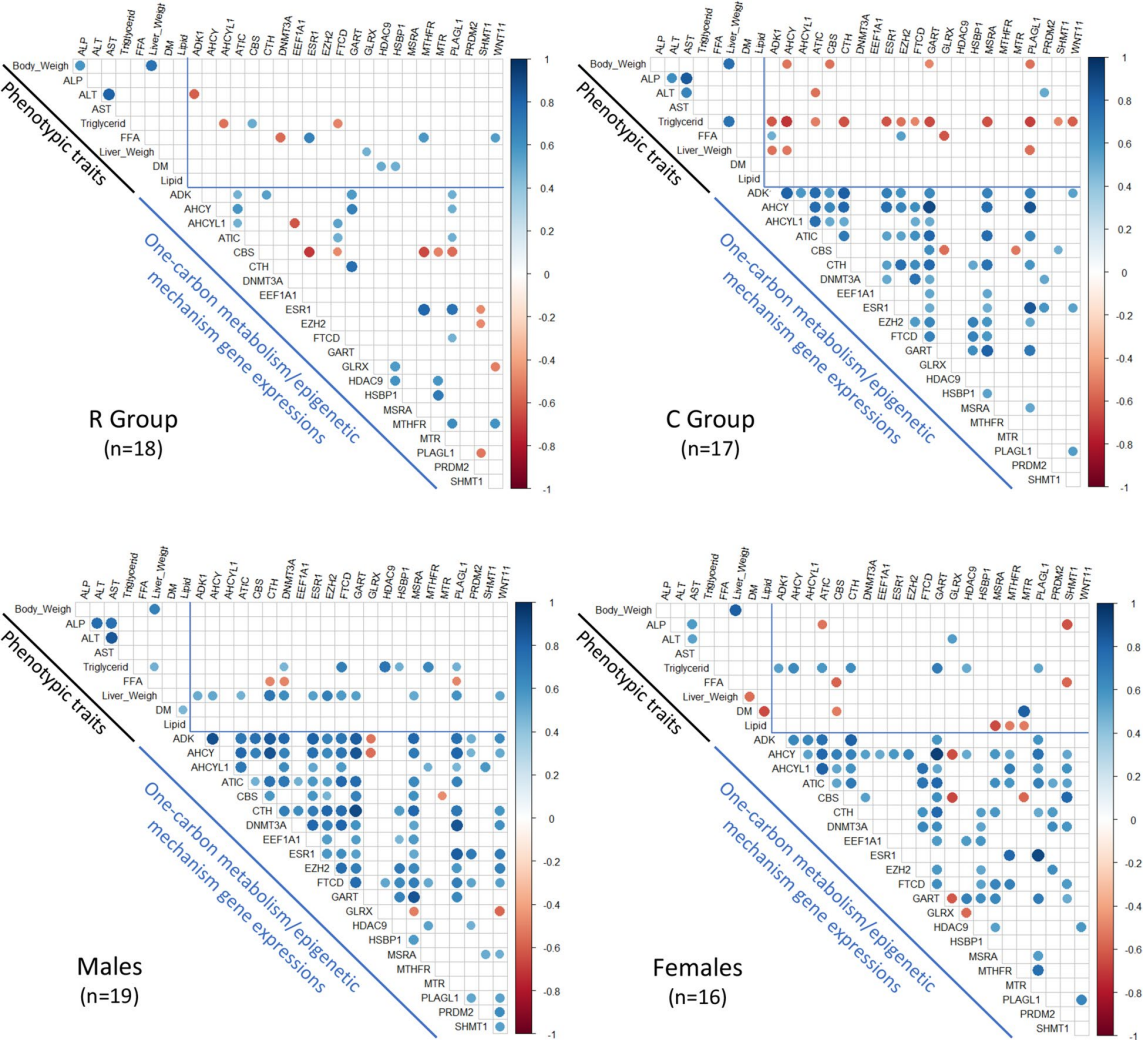
Correlation matrices between the gene expression of the 22 differential genes and the phenotypic traits of the ducklings. The correlation matrices were plotted for the R group (*n* = 18), the C group (*n* = 17), and the males (*n* = 19) and the female ducklings (*n* = 16) separately. The phenotypic traits are body weight, liver weight, percentages of liver lipids (Lipid) and liver dry mater (DM), plasma activities of ALP, ALT and AST and plasma concentrations of triglycerides (Triglycerid) and free fatty acids (FFA). The color scale indicates the strength of the correlation; blue for a positive correlation and red for a negative one. Only the significant correlations (with a *P*-value < 0.05) were plotted. For the genes, the imputed but not qqnorm transformed, normalized relative expressions were used and for the phenotypic data the raw values were used

The phenotypic traits were measured in a previous study on the same newly hatched mule ducklings [31]. There was body weight, liver weight, percentages of liver lipids and liver dry mater (DM), plasma activities of alkaline phosphatase (ALP), alanine aminotransferase (ALT) and aspartate aminotransferase (AST), triglyceride (TG) and free fatty acid (FFA) levels (Table 2A). We thus could look for correlations between the hepatic mRNA levels of the 22 differential genes and the phenotypic traits of the ducklings, first in the R and C groups and then in males and females (upper part of the correlation matrices in Fig. 2).

**Table 2:** Effects of maternal dietary Met restriction on duckling traits (from Bodin et al., [31]) and mRNA abundance of 16 DEGs mostly involved in energy metabolism (from Sécula et al., [32])

A	n	R group	n	C group	P_Diet_	P_sex_	P_interaction_
**Phenotypic Trait**		Mean ± SD		Mean ± SD			
Body weight (g)	180	33.0 ± 0.9	190	35.2 ± 0.9	< 0.001	NS	NS
Liver weight (g)	28	1.51 ± 0.08	21	1.40 ± 0.11	NS	0.001	NS
Liver: BW (%)	28	4.30 ± 0.17	21	3.92 ± 0.20	0.07	0.06	NS
Liver lipids (%)	28	17.23 ± 1.43	19	17.67 ± 1.44	NS	NS	NS
Liver dry matter (%)	28	41.10 ± 0.73	20	41.21 ± 1.10	NS	NS	NS
Plasma Glucose (mMol/L)	23	16.39 ± 1.88	26	10.63 ± 2.38	0.03	NS	NS
Plasma FFA (mMol/L)	28	0.27 ± 0.05	27	0.55 ± 0.05	0.01	0.07	NS
Log Plasma TG	27	0.55 ± 0.19	27	- 0.09 ± 0.21	0.01	0.01	NS
Log Plasma ALP	28	5.36 ± 0.09	24	5.62 ± 0.10	0.07	< 0.001	NS
Log Plasma ALT	28	2.90 ± 0.09	23	3.32 ± 0.09	0.002	0.01	NS
Log Plasma AST	27	4.42 ± 0.19	27	4.69 ± 0.21	NS	0.006	NS
B	n	R group	n	C group	P_Diet_	P_sex_	P_interaction_
**Hepatic Gene Expression**		LsMeans ± SD		LsMeans ± SD			
*BCL2A1*	18	0.34 ± 0.21	17	− 0.43 ± 0.21	0.03	0.13	NS
*COX2*	18	− 0.55 ± 0.24	17	0.77 ± 0.28	0.01	0.93	NS
*CYTB*	18	− 0.6 ± 0.28	17	0.52 ± 0.33	0.02	0.54	NS
*ELOVL6*	18	0.51 ± 0.27	17	−0.5 ± 0.31	0.01	< 0.01	NS
*ENO1*	18	−0.54 ± 0.24	17	0.51 ± 0.27	0.01	0.81	NS
*GPAM*	18	0.39 ± 0.16	17	−0.52 ± 0.17	< 0.01	< 0.01	NS
*MTTP*	18	−0.48 ± 0.21	17	0.55 ± 0.22	0.01	0.87	NS
*NDUFA4*	18	−0.61 ± 0.19	17	0.64 ± 0.19	< 0.01	0.98	NS
*NDUFB6*	18	−0.4 ± 0.22	17	0.42 ± 0.22	0.03	0.81	NS
*PGK1*	18	0.49 ± 0.25	17	−0.46 ± 0.27	0.01	*0.07*	NS
*PGM1*	18	0.53 ± 0.15	17	−0.65 ± 0.15	< 0.01	< 0.01	NS
*PPARG*	18	0.47 ± 0.22	17	−0.5 ± 0.24	0.03	0.81	NS
*PPARGC1B*	18	−0.43 ± 0.23	17	0.43 ± 0.23	0.03	0.98	NS
*PRKAA1*	18	−0.49 ± 0.2	17	0.4 ± 0.2	0.01	0.02	NS
*RXRA*	18	−0.78 ± 0.3	17	0.51 ± 0.24	0.01	0.11	NS
*UGDH*	18	0.48 ± 0.21	17	−0.53 ± 0.22	0.01	0.42	NS
*BMF* ^δ^	18	−0.48 ± 0.3	17	0.46 ± 0.34	0.06 ^δ^	0.18	NS
*PPARA* ^δ^	18	−0.35 ± 0.22	17	0.33 ± 0.22	0.10 ^δ^	0.23	NS

A: Numbers, means, and standard errors of the measured traits as well as the significance of the differences between means are given. The P-value of the diet effect, the sex effect and their interaction are presented. *P*-values < 0.05 were considered significant. B: The 16 genes differently expressed between the two diet groups (PDiet < 0.05) are listed as well as and 2 genes which tended to be differentially expressed (PDiet < 0.1; noted with a delta (^δ^)). Numbers, LS-Means, and standard deviations of the gene expression as well as the significance of the effects are given

Regarding the comparison between the matrices of correlations in the R and C groups, we observed that the maternal dietary methionine restriction led to important changes in their duckling liver. First of all, the number of significant correlations (*P* <  0.05) between the phenotypic traits and the level of expression of the 22 differential genes was 2 times lower for the R group than for the C group. For example, body weight was not correlated with any genes in the R group, whereas it was negatively correlated with the expression level of 4 genes (*AHCY*, *CBS*, *GART* and *PLAGL1*) in the C group. The plasma TG level showed even more alterations in the R group samples. Indeed, it showed only 3 correlations (2 negative ones with *AHCYL1* and *FTCD* and a positive one with *CBS*) in the R group, whereas it was negatively correlated with the expression of 12 genes in the C group, the only correlation common to both groups being with the *FTCD* gene. These results enlightened a strong effect of the maternal methionine deficiency on the number and the nature of the correlations between the expression levels of the 22 differential genes and the phenotypic traits of their offspring.

Regarding the comparison between the two sexes, the two correlation matrices differed. For instance, liver weight did not correlate with any of the differential genes in females, whereas it was positively correlated with the expression of 12 genes in males. The results also showed that ALP activity was negatively correlated with the expression level of 2 genes (*ATIC* and *SHMT1*) and that ALT activity was positively correlated with *GLRX* expression in females only. These results showed that the maternal diet affected the offspring liver gene expression in a sex-dependent way.

### Maternal methionine deficiency altered the number and nature of the correlations between the expression levels of the 22 differential genes

The Fig. 2 shows the correlation matrices of the hepatic mRNA levels of the 22 differential genes (lower part of the matrices), first in the R and C groups and then in males and females.

The very first observation was that in the ducklings of the control group (C group), the majority of the correlations were positive between the 22 differential genes. Only the *CBS* gene showed 2 negative correlations with the *MTR* and *GLRX* genes which is consistent with the opposite representation of these genes on the variable plot of the PCA (Fig. 1B). The second observation was that the number of significant correlations between the 22 differential genes was much lower in the R group compared to the C group. The expression level of *GART*, for example, showed 14 positive correlations with other differential genes in the C group, whereas it conserved only 3 of these positive correlations in the R group (with *ADK*, *AHCY* and *CTH*). These results enlightened a strong effect of the maternal methionine deficiency on the correlations between the expression levels of the 22 differential genes and thus on the liver one-carbon metabolism in their offspring.

When comparing the correlations matrices of the two sexes, we found significant differences. *Wnt11*, for example, was positively correlated with only 2 other differential genes (*HDAC9* and *PLAGL1*) in females, whereas it was positively correlated with 11 genes (*ADK1, AHCY, CTH, DNMT3A, ESR1, EZH2, FTCD, MSRA, PLAGL1, PRDM2* and, *SHMT1*) and negatively correlated with 1 gene (*GLRX*) in males. These results showed that the maternal Met-restricted diet altered the expression of genes involved in the liver one-carbon metabolism in their offspring in a sex-dependent way.

The correlation matrices between the differentially expressed genes and phenotypes and for the DEG between them were also constructed for the four duckling subgroups (MR, MC, FR and FC) and are presented in Additional Fig. 1. Quickly, the results showed that the correlation matrices of the 4 subgroups differed from each other. Indeed, the number of significant correlations decreased in the R group ducklings (MR versus MC and FR versus FC) and the correlation matrices differed between the 2 sexes (MR versus FR and MC versus FC). These observations enlightened the fact that the liver gene expression and metabolisms differ between the 4 subgroups.

### Maternal methionine deficiency altered the correlations between the expression levels of differential genes involved in one-carbon metabolism and epigenetic mechanisms and those involved in energy metabolism

**Fig. 3:**
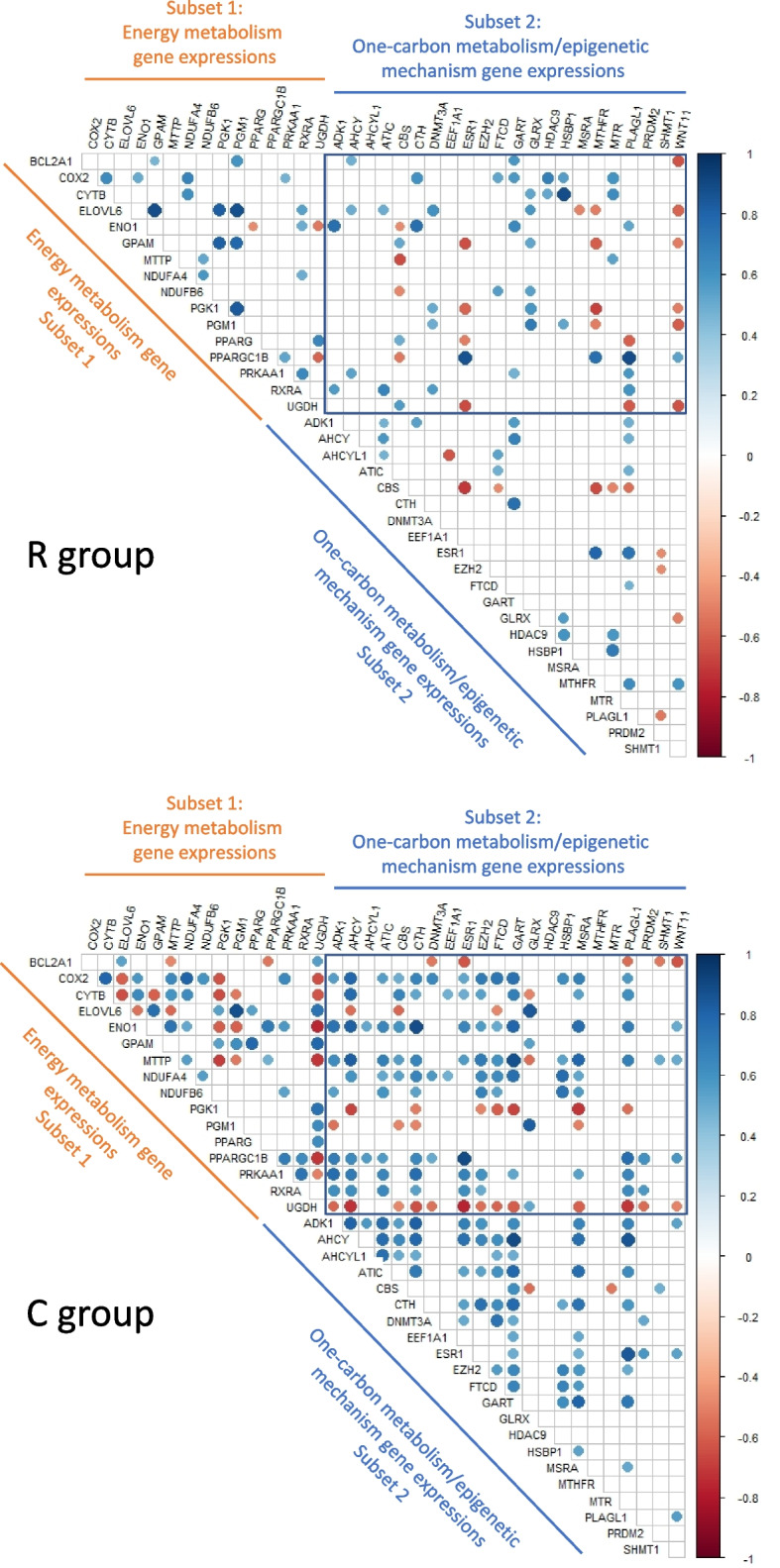
Correlation matrices between the gene expression of the two subsets of genes. The correlation matrices were plotted for the R group (*n* = 18) and the C group (*n* = 17). The group of differential genes involved in energy metabolism is referred as “Subset 1” and the group of differential genes identified in this study and mostly involved in one carbon metabolism and epigenetic mechanisms is referred as “Subset 2”. The color scale indicates the strength of the correlation; blue for a positive correlation and red for a negative one. Only the significant correlations (with a *P*-value < 0.05) were plotted. The square represents the correlations between the 16 DEGs of energy metabolism and the 22 differential genes of the one-carbon metabolism and epigenetic mechanisms. The imputed but not qqnorm transformed, normalized relative expressions were used

In addition to the phenotypic traits [31], we also recently reported the hepatic mRNA abundance of 100 genes involved in the energy metabolism in the livers of the same ducklings and we could thus identify 16 DEGs between the R and C groups [32] (Table 2B). We therefore looked for correlations between the 22 differential genes for one-carbon metabolism and epigenetic mechanisms on the one hand and the 16 DEGs previously identified for energy metabolism on the other hand. The results are given in Fig. 3. In order to simplify the description of the results, we will refer to the subset of 16 DEGs identified previously and mostly involved in energy metabolism as “Subset 1” and the subset of 22 differential genes identified in this study and mostly involved in one-carbon metabolism and epigenetic mechanisms as “Subset 2”.

Again, the striking fact is a much lower number of correlations observed between the two subsets of genes, in the R group ducklings compared to the ones of the C group, this was particularly the case for *COX2, ENO1* and *UGDH* from Subset 1 for example. On the contrary, in ducklings from the C group, *PPARG* from Subset 1 showed no correlations with differential genes from Subset 2 whereas it showed 3 correlations with genes from Subset 2 in R group ducklings. Similarly, in the C group ducklings, *MTHFR* from Subset 2 showed no correlations with differential genes from Subset 1 whereas it showed 4 negative correlations (*ELOVL6*, *GPAM*, *PGK1* and *PGM1*) and 1 positive correlation (*PPARGC1B*) with genes from Subset 1 in the R group ducklings.

If focusing on the differential genes involved in epigenetic mechanisms (*DNMT3A*, *EZH2*, *HDAC9* and *PRDM2*), we can highlight that the correlations differed between the two groups of ducklings, both for the correlations with the differential genes related to energy metabolism (subset 1) and with the differential genes related to one-carbon metabolism (subset 2). This was particularly true for *EZH2*, which showed 10 correlations - 8 of which were positive - with the energy metabolism genes in the C group, whereas it did not show any correlations in the R group. The same *EZH2* gene showed 4 positive correlations with the one-carbon metabolism genes in the C group, whereas it showed none in the R group.

More generally, it should be noted that many positive correlations were observed between the differential genes of the 2 metabolisms (subset1 and subset2) in the ducklings of the control group, whose mothers received an adequate supply of methionine. In contrast, the number of positive correlations between the genes of the 2 metabolisms decreases significantly in the livers of ducklings whose mothers were fed methionine-deficient diet. Altogether, these results showed that the maternal Met-restricted diet altered correlations between the two groups of genes i.e. links between the two studied metabolisms.

## Discussion

It is well established that one-carbon metabolism, which includes the methionine and folate cycles, provides methyl groups for epigenetic mechanisms such as DNA, histone or miRNA methylation as well as for a number of metabolic pathways as reviewed by Clare and colleagues [15]. In this work, we wanted to know if a maternal deficiency of methionine, which is a methyl group donor, could have altered the hepatic level of transcription of genes involved in one-carbon metabolism in the offspring. We also added to the study a few genes involved in epigenetic control of gene expression (*DNMT3A*, *EZH2*, *HDAC9* and *PRDM2*), in cellular responses to stress (*HSBP1* and *EEF1A1*), or encoding transcription factors (*ESR1* and *PLAGL1*) or signaling proteins (*WNT11*).

**Fig. 4:**
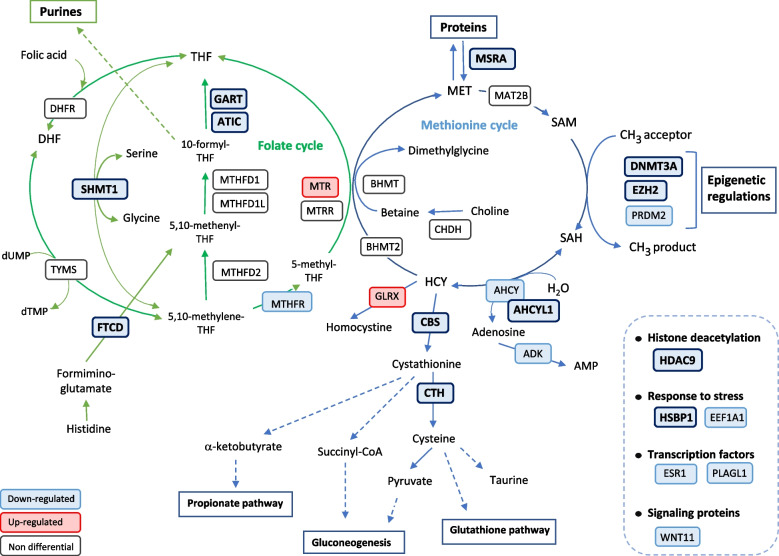
Role of the 22 differential genes assigned to one-carbon metabolism and epigenetic mechanisms and their regulation in newly hatched ducklings from the R group (adapted from Clare et al. [15],). Only the genes that have been studied in this work are mentioned. The folate cycle is represented in green and the methionine cycle is represented in blue. The differential genes for the maternal diet that are downregulated or upregulated when comparing the R group ducklings to the C group ducklings are in blue and red, respectively. The 12 DEGs (with a Diet *p*-value (BH) <  0.05) are in bold whereas the 10 ones which tend to be differently expressed between the two diet groups (with a Diet p-value (BH) <  0.10) are not. They were all downregulated in the R group samples when compared to C group samples, except *GLRX* and *MTR* that were upregulated. ADK, Adenosine Kinase; AHCY, Adenosylhomocysteinase; AHCYL1, Adenosylhomocysteinase Like 1; ATIC, 5-Aminoimidazole-4-Carboxamide Ribonucleotide Formyltransferase/IMP Cyclohydrolase; BHMT/2, Betaine--Homocysteine S-Methyltransferase/2; CBS, Cystathionine Beta-Synthase; CHDH, Choline Dehydrogenase; CTH, Cystathionine Gamma-Lyase; DHFR, Dihydrofolate Reductase; DNMT3A, DNA Methyltransferase 3 Alpha; EZH2, Enhancer Of Zeste 2 Polycomb Repressive Complex 2 Subunit; FTCD, Formimidoyltransferase Cyclodeaminase; GART, Phosphoribosylglycinamide Formyltransferase; GLRX, Glutaredoxin; MAT2B, Methionine denosyltransferase 2B; MSRA, Methionine Sulfoxide Reductase A; MTHFD1/2, Methylenetetrahydrofolate Dehydrogenase1/2; MTHFD1L, Methylenetetrahydrofolate Dehydrogenase (NADP+ Dependent) 1 Like; MTHFR, Methylenetetrahydrofolate Reductase; MTR, 5-Methyltetrahydrofolate-Homocysteine Methyltransferase; MTRR, 5-Methyltetrahydrofolate-Homocysteine Methyltransferase Reductase; PLAGL1, PLAG1 Like Zinc Finger 1; PRDM2, PR/SET Domain 2; SHMT1, Serine Hydroxymethyltransferase 1; TYMS, Thymidylate Synthetase; WNT11, Wnt family member 11. DHF, dihydrofolate; dTMP, thymidine monophosphate; dUMP, deoxyuridine monophosphate; HCY, homocysteine; MET, methionine; SAH, S-adenosylhomocysteine; SAM, S-adenosylmethionine; THF, tetrahydrofolate. HDAC9, Histone Deacetylase 9; EEF1A1, Eukaryotic translation elongation factor 1 alpha 1; HSBP1, Heat Shock factor binding protein 1; ESR1, estrogen receptor 1

The Fig. 4 provides an overview of the role of the 22 differential genes (both the 12 DEGs with a *p*-value < 0.05 and the 10 genes which tended to be differential with a 0.05 < p-value < 0.10) and their regulation in newly hatched ducklings from the R group when compared to the C group. The maternal dietary Met deficiency led to either the downregulation (*MTHFR*, *ATIC*, *GART* and *SHMT1*) or the upregulation (*MTR)* of genes involved in the folate cycle, while other genes remained unaffected by the maternal nutrition (*DHFR*, *TYMS*, *MTHFD1, MTHFD2*, *MTHFD1L* and *MTRR*). *FTCD*, whose product serves to conduit one-carbon units from formiminoglutamate to the folate pool, was also downregulated. Similarly, some genes belonging to the methionine cycle were not differentially expressed (*BHMT*, *BHMT2*, *MAT2B*, *CHDH*) whereas *AHCY*, whose product regulates the intracellular concentration of S-adenosylhomocysteine (SAH), was downregulated. Moreover, *AHCYL1* and *ADK*, which encode proteins that are involved in the conversion of SAH into homocysteine (HCY) and adenosine and then to adenosyl mono-phosphate (AMP) [15], were also downregulated. *GLRX* that encodes a protein which metabolizes two homocysteine molecules to homocystine was upregulated in ducklings from the R group.

We previously showed that plasma parameters were affected in newly hatched ducklings from Met-restricted dams. Thus, their glucose and triglyceride (TG) concentrations were higher while their free fatty acid (FFA) levels as well as alanine transaminase (ALT) activity were decreased suggesting an alteration in their hepatic energy metabolism [31]. Moreover, we also reported hepatic differential expression of genes either involved in different pathways related to energy metabolism such as glycolysis, lipogenesis or electron transport or encoding nuclear receptors such as PPARGC1B, PPARG and RXRA [32] between the two groups of ducklings. Thus, taken together, our results showed that maternal methionine deficiency had an impact on the mRNA abundance of genes involved in both one-carbon metabolism, epigenetic mechanisms and energy metabolism in the liver of the newly hatched offspring, in line with the observed changes in their phenotypic traits.

Our observations are in accordance with the literature where the role of methyl group balance to maintain normal liver function and direct interplays between one-carbon metabolism and liver injury such as non-alcoholic fatty liver disease has been reviewed [15, 33, 34]. Indeed, SAM is utilized in methyl-group transfer for the synthesis of phosphatidylcholine which is the major phospholipid component of lipoproteins and is required for HDL and cholesterol synthesis in the liver as well as for the synthesis of VLDL, which export lipids from the liver [35]. SAM depletion may thus contribute to insufficient hepatic phosphatidylcholine synthesis and inadequate hepatic TG export, leading to intrahepatic TG accumulation [36]. SAM is also utilized in methyl-group transfer to DNA by DNA methyltransferases and to the side chains of lysine and arginine residues of histones by either histone lysine N-methyltransferases or histone arginine N-methyltransferases whereas level of histone acetylation is modulated by histone acetylases and histone deacetylases. Therefore, a lower activity of hepatic one-carbon metabolism may lead to aberrant DNA and histone methylation patterns and thus may modify chromatin remodeling and gene expression leading to altered hepatic transcriptome and metabolism [21, 37]. Indeed, the impact of maternal methyl donor deficiency on offspring hepatic DNA methylation, transcriptome or metabolism has already been reported in several mammalian models [17, 38–43]. In chicken, *in ovo* injection of betaine has been reported to affect hepatic cholesterol metabolism [26], DNA and histone methylation and expressions of *PPARα* and *CPT1α* in newly hatched chicks [27]. Beside this, maternal dietary betaine supplementation changed hepatic transcriptome and also increased hepatic protein contents of BHMT and DNMT1 and decreased the hepatic cholesterol content in offspring as already mentioned [28, 44].

In the present work, and beside its impacts on the mRNA abundance of genes involved in one-carbon metabolism, the maternal dietary Met deficiency reduced expression of genes involved in epigenetic mechanisms such as *HDAC9* whose product is related to histone deacetylation. Three genes encoding methyltransferases were also downregulated in the ducklings from the Met-restricted dams; *DNMT3A* involved in DNA methylation, and *EZH2* and *PRDM2* which are both SAM-dependent histone methyltransferases [45] involved in chromatin remodeling regulating gene expression [46]. The maternal dietary Met deficiency also downregulated the expression of 2 genes involved in cellular response to stress (*EEF1A1* and *HSBP1*). On the one hand, in case of lipid accumulation in hepatocytes, EEF1A-1 (Eukaryotic elongation factor 1A-1) responds to endoplasmic reticulum stress and promotes cell death [47, 48]. On the other hand, HSBP1 by binding to HSF1 (heat shock factor 1), which is a transcription factor involved in the heat-shock response, acts as a negative regulator of heat shock response [49]. Finally, the maternal dietary Met deficiency also downregulated the expression of 2 genes encoding transcription factors (*ESR1*, *PLAGL1*) and one gene encoding a signaling protein (*WNT11*). Again, the three of them were downregulated. Altered ESR1 function is associated with obesity and metabolic dysfunction in humans [50] and it was reported to be critical for regulation of lipid metabolism in mice [51] where it regulates the synthesis of cholesterol transport proteins, enzymes for lipoprotein remodeling, and receptors for cholesterol [52]. *PLAGL1*, for its part, encodes a zinc finger protein that plays roles as transcription factor as well as cofactor of nuclear receptors and is considered a tumor suppressor factor which regulates apoptosis and cell cycle arrest [53, 54]. The expression of *PLAGL1* gene is controlled through CpG methylation and histone deacetylation [53], and DNA methylation of *PLAGL1* was associated with maternal folate levels and birth weight in human [55]. Interestingly, PLAGL1 was also reported to regulate the transcription of *PPARG* (Peroxisome Proliferator Activated Receptor Gamma) [54] which is a master regulator of lipogenesis which has been described to promote lipid storage in the liver. Finally, the WNT gene family encodes secreted signaling glycoproteins which bind to receptors to initiate a signaling cascade thus regulating signaling events. They are implicated in developmental processes such as regulation of cell fate and patterning during embryogenesis [56] and involved in proliferation and terminal differentiation of hepatic progenitors, hepatic metabolic zonation and regeneration, and in hepatic metabolism [57–59]. Consequently, dysregulation of Wnt signaling may lead to chronic metabolic diseases -including NAFLD- and cancers [60–62]. Regarding Wnt11, it is expressed during hepatic differentiation [59] and contributes to liver zonation [63]. Moreover, its expression was reported to be increased in a mouse model of methionine-choline deficient diet (MCDD)-induced NASH which is a severe form of NAFLD [64].

In conclusion, this avian model showed that maternal methyl donor deficiency had an impact on the mRNA abundance of 22 genes mostly involved in one-carbon metabolism or epigenetic mechanisms and on the mRNA abundance of 16 genes involved in energy metabolism in the liver of the newly hatched offspring, in line with the previously observed changes in their phenotypic traits. This work also showed strong correlations i.e. strong links between the transcript level of genes involved in one-carbon metabolism or epigenetic mechanisms and the transcript level of genes involved in energy metabolism. This is in accordance with previous publications highlighting the links between one-carbon metabolism, epigenetic mechanisms and energy metabolism in mammals [21, 24]. Future work will focus on the impacts of this maternal dietary methionine restriction on the liver metabolism of adult offspring at 14 weeks of age -after a 12 days period of overfeeding- for which a reduction in the fatty liver weight was observed (unpublished data). This will make it possible to evaluate the long-term impacts of the maternal diet at a molecular level and confirm a nutritional programming of the hepatic energy metabolism in offspring of both sexes.

## Methods

The methods described in this work are exactly the same as those described in a recent article by Sécula et al. [32] because the two articles report to two parts of the same study.

### Experimental design and sample collection

Experimental procedures and animal care were conducted in compliance with the European Communities Council Directive 2010/63/EU. The protocol and procedures were approved by the French Minister of Higher Education, Research and Innovation (authorization APAFIS#1847-2015092213418825v2). The experiment was conducted at the Ducks and Goose Experimental Facility – INRAE, UEPFG, (Benquet, France) that received the accreditation number B40–037-1.

The experimental design has already been described twice [31, 32]. Briefly, 60 female common ducks were fed an adequate level of Met until 10 weeks of age and were then divided into two groups before being fed experimental diets from 10 to 51 weeks of age. The R group was fed Met-restricted experimental diets containing 0.25% of Met, while the C group was fed control experimental diets containing 0.40% Met that meets the Met requirement of 0.40–0.45% for laying ducks [65–68]. For duckling production, 2 artificial inseminations per week were performed between 34 and 36 weeks of age, using the semen of 15 Muscovy drakes fed commercial diets. The eggs were incubated for 28 days at 37.6 °C and 60% mean relative humidity throughout the incubation period (Sologne incubator, La Nationale, Briaire, France). They were then placed in a hatchery (Bretagne hatchery, La Nationale, Briaire, France) for 4 days at 37.3 °C and 80% of mean relative humidity. Mule ducklings that were the offspring of females in R and C groups were then assigned to R and C groups, respectively. Duckling phenotypic traits were recorded at hatching on 180 and 190 ducklings from R and C groups respectively, as already reported [31]. In addition, a total of 58 ducklings were sacrificed by cervical dislocation at hatching without being fed prior to sacrifice (12 females and 16 males from the C group and 15 females and 15 males from the R group). Their liver weight was recorded and the livers of 8 females and 13 males from the C group and 15 females and 15 males from the R group were immediately immersed in liquid nitrogen before being transferred to a freezer at − 80 °C.

### RNA extraction and reverse transcription

Frozen liver samples from newly hatched ducklings of both sexes and from both diet groups (10 males and 8 females in the C group and 10 males and 10 females in the R group) were ground using a Retsch grinder at 30 Hz for 45 seconds in liquid nitrogen. Next, 80–100 mg of tissue powder was processed as previously described [69] for RNA extraction and purification using the TRIzol® method (Invitrogen, California, USA) followed by a Nucleospin RNA kit column (Macherey Nagel, France) and following the manufacturer’s instructions. DNAse treatment on the column was performed with 20 μl of rDNAse (Macherey Nagel) and 80 μl of reaction buffer for 20 min to avoid DNA contamination, as recommended [70, 71]. Total RNA was quantified using the NanoDrop 8000 spectrophotometer (Thermo Fisher, Illkirch, France) and stored at − 80 °C. Its integrity was checked by electrophoresis and using an Agilent 2100 bioanalyzer, with the RNA 6000 Nano Lab Chip kit (Agilent Technologies, Massy, France). Reverse transcription was performed immediately after quality control evaluation, and the same amount of total RNA was used for all experimental samples, as recommended [72]. The reaction used SuperScriptTM II reverse transcriptase (Invitrogen, California, USA), RNasin® ribonuclease inhibitor (Promega Corporation, USA) and oligo (dT)15 (Sigma Aldrich, France). The cDNAs were then diluted in RNase-free water and stored at − 80 °C.

### Primer design and qPCR validation

We targeted 70 genes known to be related to one-carbon metabolism or epigenetic mechanisms and their sequences were obtained from the NCBI [73] and/or Ensembl [74] databases either in *Anas platyrhynchos* if available or in *Gallus gallus*. The two primers used for each gene (Additional Table 2) were each designed on either side of an intron, and for a hybridization temperature of 60 °C, using either Primer3Plus [75] or LightCycler® Probe Design 2.0 software (Roche Applied Science). Primer sequences were blasted to databases to confirm that they were specific to the gene in question and PCR products were subjected to 2% agarose gel electrophoresis to confirm amplicon size. Next, primer pairs showing a specific band and the absence of primer dimers were selected for qPCR testing, using SYBR green fluorescence detection (Applied Biosystems) and a QuantStudio6 (Thermo Fisher Scientific). Each primer pair was tested on four serial dilutions of a cDNA pool (cDNA from all animals used in the study) to obtain a standard curve and to check PCR efficiency, with each point being performed in duplicate. The conditions were: 50 °C for 2 min, denaturation at 95 °C for 10 min, 40 cycles of 15 s at 95 °C and 1 min at 60 °C. A gradual increase in temperature from 60 °C to 95 °C was added to analyze the melting curves and detect primer dimers. Cq values and PCR efficiency were obtained directly from QuantStudio Real-Time PCR v1.3 software.

### Identification of potential reference genes, quantitative PCR and gene expression analysis

The stability of expressions of 9 genes (*ALB*, *GAPDH*, *HMBS*, *HPRT1*, *NDUFA10, POLA1*, *RPS13, TBP* and *TUBA1C*) was tested in the livers of newly hatched mule ducklings as already described [32]. These 9 primer pairs were tested by qPCR on liver cDNA from 8 ducklings of both sexes and both maternal diet groups (R and C groups) with SYBR green fluorescence detection, on a QuantStudio6. The selection of the 5 most stable genes (*GAPDH*, *HMBS*, *NDUFA10*, *RPS13*, *TBP*) was performed with the SLqPCR package on RStudio [76]. These 5 genes are highlighted in dark grey in Additional Table 2.

Gene expression was quantified using 96.96 Dynamic Array Integrated Fluidic Circuits (IFCs) and the Fluidigm BioMark HD system, as described previously [77]. The entire experiment was conducted on 168 liver samples (38 samples of newly hatched duckling livers for the current study and 130 samples of older duck livers) and a total of 170 genes, either targeting one-carbon metabolism (for this study, 70 genes, called “Subset 2”) or playing a role in energy metabolism (for a previously published work, 100 genes [32], called “Subset 1”). As the technology used did not allow for all samples and genes to be analyzed on the same chip, care was taken to randomize the samples on two chips and the genes in each specific target amplification (STA), thus making a total of four chips. In this paper we report only the study of the expression of genes involved in one-carbon metabolism (Subset 2) in the liver of newly hatched ducklings. For each of the 4 arrays, a 14-cycle STA was performed on the cDNA samples, a calibrator sample (a pool of 38 cDNA samples), a cDNA pool of 168 cDNA samples in fivefold dilutions (to determine PCR amplification efficiency), a duck genomic DNA control, an internal control (human genomic DNA) and a negative control (TE).

The resulting cDNA samples were then processed as previously reported [32, 77]. Fluidigm Digital PCR Analysis software (version 4) was used to analyze the data, using the linear (derivative) baseline correction method and the automatic (global) cycle threshold (Ct) method. Pre-processing of the data was carried out by removing cycle threshold (Ct) values recorded from amplifications whose melting curves showed either an abnormal Tm (melting temperature), double peaks (corresponding to a mixture of expected and aberrant PCR products), or a high baseline. The slope of the standard curve obtained with serial dilutions of the 168 cDNA pool was used to measure the PCR efficiency (E) for each gene and genes with less than three dilution points were eliminated. Finally, all genes with efficiencies greater than 2.2 or less than 1.7 were also eliminated from the analysis. Then, the relative expression (RE(i,j)) for each gene (i) and sample (j) was calculated as proposed by Pfaffl [78]: RE(i,j) = Eff(i)^(Cq(i,cal) - Cq(i,j)) where Cq(i,cal) is the Cq of the gene (i) determined for the calibrator sample (a pool of 38 cDNA samples). The stability of the five potential genes was assessed with the GeNorm algorithm (version 3.4) [76] and the three most stable genes were identified (*GAPDH*, *RPS13* and *TBP*). The normalization of the relative expression RE_n_(i,j) for each gene (i) and sample (j) was computed by dividing the RE(i,j) for each gene (i) and sample (j) by the geometric mean expression of these three reference genes for the sample (j), as proposed by Vandesompele et al. [76].

At the end of this analysis, 8 of the 70 genes studied and 2 of the 38 liver cDNA samples had more than 25% missing data and were removed from the study. Moreover, another cDNA sample showing outlier gene expression points in a Principal Component Analysis (PCA, not showed) was removed from the data set. The 8 deleted genes are highlighted in light grey in Additional Table 2. Thus, the current analyses were performed on the 62 remaining genes with the remaining 35 liver cDNA samples which were from 9 male and 8 female ducklings from C group and 10 male and 8 female ducklings from R group.

### Statistical analyses

For the remaining 62 genes, the few missing values were imputed within each group of similar sex and maternal diet using the imputPCA function with 3 principal components from the missMDA package of R software [79]. These normalized and imputed relative expressions were then transformed using the function qqnorm(Y)$x to make the data follow a centered reduced normal distribution and it is these transformed data that were then used to describe the data set. First, a Partial Least Square Discriminant Analysis (PLS-DA) was performed with the package MixOmics of R software [80] on the 62 genes and the individuals were plotted on the two first latent variables of the PLS-DA score plot.

ANOVAs were then conducted on the qqnorm transformed normalized relative expressions of the 62 genes using a linear mixed model fitted with ASReml software [81] as previously described [32]. This model included maternal diet, duckling sex and the interaction between them as fixed effects, and the duckling associated with its relationship matrix as a random effect. We selected as differentially expressed genes (DEGs) the genes with a significant difference - diet *P*-value < 0.05 assessed after a Benjamini-Hochberg (1995) correction [82], to account for multiple tests (diet P-value (BH)). The effect of sex (sex P-value (BH)), as well as the interaction between the effects of sex and diet (sex P-value*diet (BH)) on gene expression were also assessed. Least square means (LS means) and standard deviations were calculated for the 2 maternal diet groups (R and C groups), for the 2 sexes, and finally for the four subgroups of interest, i.e. males and females from the R group (MR and FR) and males and females from the C group (MC and FC) (Additional Table 1).

Additional analyses were performed on the DEGs for the effect of diet. First, a PCA was performed on the DEGs with the MixOmics package of the R software [80], using the qqnorm transformed normalized relative expressions. Using the Factoextra package of R, the biplot of the variables and samples of the first two principal components was obtained and then the ellipse package was used to plot the concentration ellipses around the mean points of each group with a confidence level of 0.75. Next, correlation matrices were performed with the ducklings’ phenotypic traits and the DEGs for the effect of diet, using the imputed but not qqnorm transformed normalized relative expressions, to be consistent with the phenotypic data which were also not transformed. The correlation matrices were plotted with the package Hmisc of R using the functions rcorr and corrplots [83, 84] and only the correlations with a *P*-value < 0.05 were reported.

Lastly, this study was conducted and is reported in accordance with the ARRIVE guidelines [85].

## Supplementary Information

**Additional file 1.**

## Data Availability

The datasets used and analyzed during the current study are available from the corresponding author.

## Abbreviations

ADK: Adenosine Kinase
AHCY: Adenosylhomocysteinase
AHCYL1: Adenosylhomocysteinase Like 1
ALP: Alkaline Phosphatase
ALT: Alanine Transaminase
ANOVA: Analysis of variance
AST: Aspartate aminotransferase
ATIC: 5-Aminoimidazole-4-Carboxamide Ribonucleotide Formyltransferase/IMP Cyclohydrolase
BHMT/2: Betaine--Homocysteine S-Methyltransferase/2
CBS: Cystathionine Beta-Synthase
cDNA: Complementary deoxyribonucleic acid
CHDH: Choline Dehydrogenase
Cq: Cycle threshold
Ct: Cycle threshold value
CTH: Cystathionine Gamma-Lyase
DEG: Differentially Expressed Gene
DHF: Dihydrofolate
DHFR: Dihydrofolate Reductase
DM: Dry mater
DNAse: Deoxyribonuclease
DNMT3A: DNA Methyltransferase 3 Alpha
dTMP: Thymidine monophosphate
dUMP: Deoxyuridine monophosphate
EEF1A1: Eukaryotic translation elongation factor 1 alpha 1
ESR1: Estrogen receptor 1
EZH2: Enhancer Of Zeste 2 Polycomb Repressive Complex 2 Subunit
FC: Females from the C group
FFA: Fatty acids
FR: Females from the R group
FTCD: Formimidoyltransferase Cyclodeaminase
GAPDH: Glyceraldehyde-3-phosphate dehydrogenase
GART: Phosphoribosylglycinamide Formyltransferase
GLRX: Glutaredoxin
HCY: Homocysteine
HSBP1: Heat Shock factor binding protein 1
MAT2B: Methionine denosyltransferase 2B
MC: Males from the C group
MET: Methionine
Met: Methionine
MR: Males from the R group
mRNA: Messenger ribonucleic acid
MSRA: Methionine Sulfoxide Reductase A
MTHFD1/2: Methylenetetrahydrofolate Dehydrogenase1/2
MTHFD1L: Methylenetetrahydrofolate Dehydrogenase (NADP+ Dependent) 1 Like
MTHFR: Methylenetetrahydrofolate Reductase
MTR: 5-Methyltetrahydrofolate-Homocysteine Methyltransferase
MTRR: 5-Methyltetrahydrofolate-Homocysteine Methyltransferase Reductase
PCA: Principal Component Analysis
PCR: Polymerase Chain Reaction
PLAGL1: PLAG1 Like Zinc Finger 1
PPARA: Peroxisome proliferator activated receptor alpha
PPARG: Peroxisome proliferator activated receptor gamma
PPARGC1B: PPARG Coactivator 1 Beta
PRDM2: PR/SET Domain 2
qPCR: Quantitative Real-Time PCR
RE: Relative expression
RNAse: Ribonuclease
RXRA: Retinoid X Receptor Alpha
SAH: S-adenosylhomocysteine
SAM: S-adenosylmethionine
SHMT1: Serine Hydroxymethyltransferase 1
TCA cycle: Tricarboxylic acid cycle
TE: Tris-EDTA buffer
TG: Triglycerides
THF: Tetrahydrofolate
HDAC9: Histone Deacetylase 9
Tm: Melting temperature
TYMS: Thymidylate Synthetase
WNT11: Wnt family member 11
