# Supplementary material for: Maternal dietary methionine restriction alters hepatic expression of one-carbon metabolism and epigenetic mechanism genes in the ducklings
Source: BMC Genomics. 2022 Dec 12;23:823. doi: 10.1186/s12864-022-09066-7 (PMC9746021; doi:10.1186/s12864-022-09066-7)
Supplement: Supplementary file 1 — Additional file 1. [file 12864_2022_9066_MOESM1_ESM.docx]

**Additional Table 1: Statistical results of gene expression in the liver of ducklings.**

The genes are listed according to whether they are differentially expressed (first part of the table, highlighted in dark grey; (Diet p-value (BH) < 0.05)), tend to be differentially expressed (second part of the table, highlighted in light grey; (Diet p-value (BH) < 0.10)) or are not differentially expressed for the maternal diet (white background). The genes which are differentially expressed (Sex P-value (BH) < 0.05) or tend to be differentially expressed (Sex P-value (BH) < 0.1); noted with a delta, ^δ^) depending of the sex of the duckling are in bold. For each gene, least square means (LS-Means) and standard deviations (SD) are presented for the two groups of maternal diet (R and C groups), for the two sexes, and for the four subgroups of interest, i.e. males (MR) and females (FR) in the R group and males (MC) and females (FC) in the C group. The corrected P-values with Benjamini-Hochberg procedure of the diet effect, the sex effect and their interaction are presented. The data used were the qqnorm transformed normalized relative expressions.

**Additional Table 2: Description of the 70 targeted genes and the 5 potential reference genes.**

The genes targeted in the study are related to one-carbon metabolism, epigenetic mechanisms and cellular stress, or are transcription factors. Genes showing more that 25% of missing data were removed from the study and are highlighted in light grey. The 5 potential reference genes are highlighted in dark grey. Molette and Massimino: unpublished data from Caroline Molette and William Massimino.

**Additional Figure 1: Correlation matrices of the transcript level of the 22 differential genes and the phenotypic traits in the four subgroups of ducklings.**


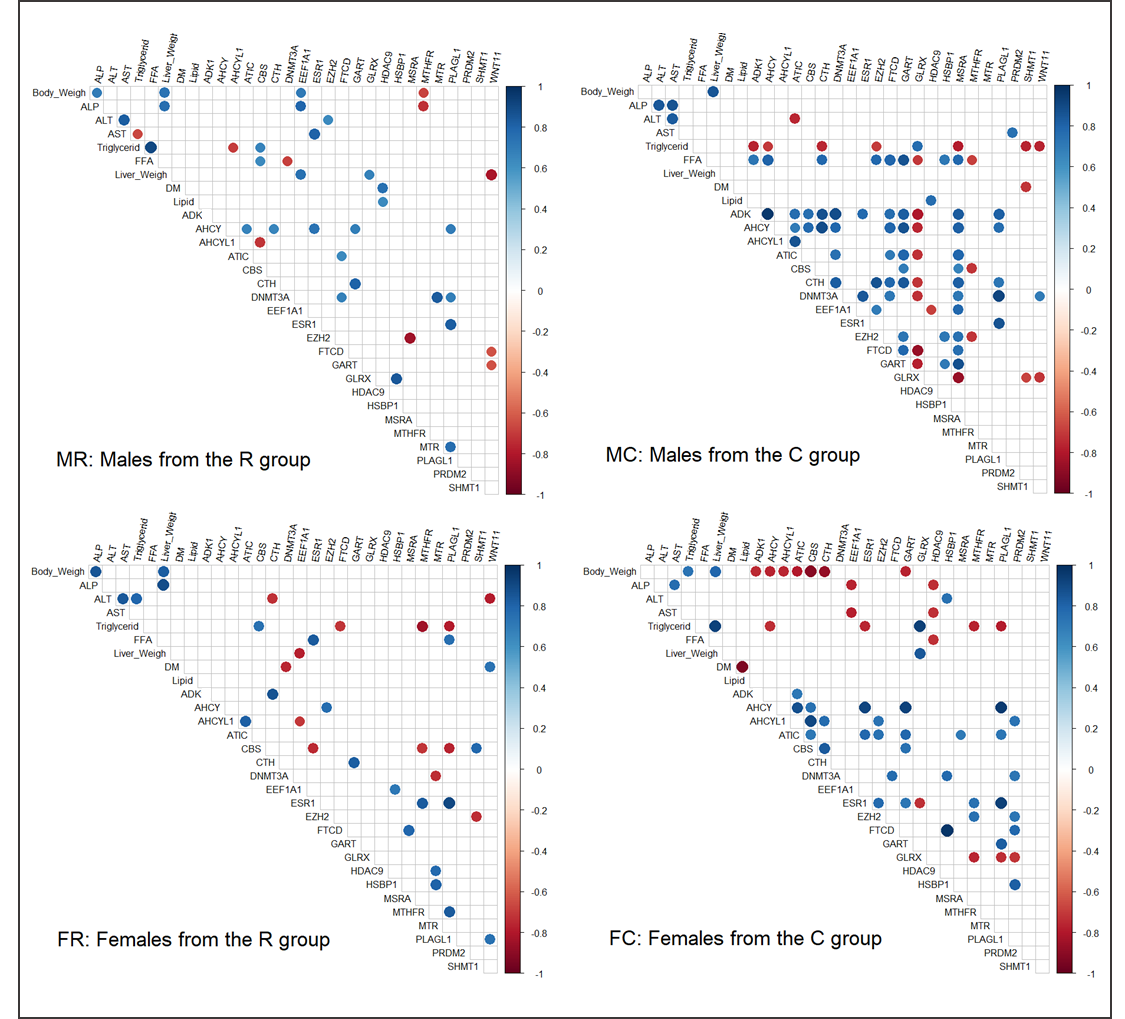


The correlation matrices were plotted for the four subgroups of ducklings i.e. males (MR; n = 10) and females (FR; n = 8) of the R group, and males (MC; n = 9) and females (FC; n = 8) of the C group. Phenotypic traits are liver weight, percentages of liver lipids and liver dry mater (DM), plasma activities of ALP, ALT and AST, triglyceride and free fatty acid (FFA) concentrations. The color scale indicates the strength of the correlation; blue for a positive correlation and red for a negative one. Only the significant correlations (with a P-value < 0.05) were plotted. For the genes, the imputed but not qqnorm transformed, normalized relative expressions were used and for the phenotypic data the raw values were used.
